# Surveillance for Waterborne Disease Outbreaks Associated with Drinking Water — United States, 2013–2014

**DOI:** 10.15585/mmwr.mm6644a3

**Published:** 2017-11-10

**Authors:** Katharine M. Benedict, Hannah Reses, Marissa Vigar, David M. Roth, Virginia A. Roberts, Mia Mattioli, Laura A. Cooley, Elizabeth D. Hilborn, Timothy J. Wade, Kathleen E. Fullerton, Jonathan S. Yoder, Vincent R. Hill

**Affiliations:** ^1^Epidemic Intelligence Service, CDC; ^2^Division of Foodborne, Waterborne, and Environmental Diseases, National Center for Emerging and Zoonotic Infectious Diseases, CDC; ^3^Division of Bacterial Diseases, National Center for Immunization and Respiratory Diseases, CDC; ^4^U.S. Environmental Protection Agency.

Provision of safe water in the United States is vital to protecting public health (*1*). Public health agencies in the U.S. states and territories* report information on waterborne disease outbreaks to CDC through the National Outbreak Reporting System (NORS) (https://www.cdc.gov/healthywater/surveillance/index.html). During 2013–2014, 42 drinking water–associated^†^ outbreaks were reported, accounting for at least 1,006 cases of illness, 124 hospitalizations, and 13 deaths. *Legionella* was associated with 57% of these outbreaks and all of the deaths. Sixty-nine percent of the reported illnesses occurred in four outbreaks in which the etiology was determined to be either a chemical or toxin or the parasite *Cryptosporidium.* Drinking water contamination events can cause disruptions in water service, large impacts on public health, and persistent community concern about drinking water quality. Effective water treatment and regulations can protect public drinking water supplies in the United States, and rapid detection, identification of the cause, and response to illness reports can reduce the transmission of infectious pathogens and harmful chemicals and toxins.

To provide information about drinking water–associated waterborne disease outbreaks in the United States in which the first illness occurred in 2013 or 2014 (https://www.cdc.gov/healthywater/surveillance/drinking-surveillance-reports.html), CDC analyzed outbreaks reported to the CDC Waterborne Disease and Outbreak Surveillance System through NORS (https://www.cdc.gov/nors/about.html) as of December 31, 2015. For an event to be defined as a waterborne disease outbreak, two or more cases must be linked epidemiologically by time, location of water exposure, and illness characteristics; and the epidemiologic evidence must implicate water exposure as the probable source of illness. Data requested for each outbreak include 1) the number of cases, hospitalizations, and deaths; 2) the etiologic agent (confirmed or suspected); 3) the implicated water system; 4) the setting of exposure; and 5) relevant epidemiologic and environmental data needed to understand the outbreak occurrences and for determining the deficiency classification.^§^ One previously unreported outbreak with onset date of first illness in 2012 is presented but is not included in the analysis of outbreaks that occurred during 2013–2014.

Public health officials from 19 states reported 42 outbreaks associated with drinking water during the surveillance period (Table 1) (https://www.cdc.gov/healthywater/surveillance/drinking-water-tables-figures.html). These outbreaks resulted in at least 1,006 cases of illness, 124 hospitalizations (12% of cases), and 13 deaths. At least one etiologic agent was identified in 41 (98%) outbreaks. Counts of etiologic agents in this report include both confirmed and suspected etiologies, which differs from previous surveillance reports. *Legionella* was implicated in 24 (57%) outbreaks, 130 (13%) cases, 109 (88%) hospitalizations, and all 13 deaths (Table 1). Eight outbreaks caused by two parasites resulted in 289 (29%) cases, among which 279 (97%) were caused by *Cryptosporidium*, and 10 (3%) were caused by *Giardia duodenalis*. Chemicals or toxins were implicated in four outbreaks involving 499 cases, with 13 hospitalizations, including the first reported outbreaks (two outbreaks) associated with algal toxins in drinking water.

**TABLE 1:** Waterborne disease outbreaks associated with drinking water (N = 42), by state/jurisdiction and month of first case onset — Waterborne Disease and Outbreak Surveillance System, United States, 2013–2014

State/ Jurisdiction	Month	Year	Etiology*	Predominant illness^†^	No. of cases	No. of hospitalizations^§^	No. of deaths^¶^	Type of water system**	Water source	Setting
Alaska	Aug	2014	*Giardia duodenalis* ^††^	AGI	5	0	0	Community	River/Stream	Community/Municipality
Arizona	Jan	2014	Norovirus (S)	AGI	4	0	0	Transient, noncommunity	Unknown	Camp/Cabin Setting
Florida	Sep	2013	*L. pneumophila* serogroup 1	ARI	4	4	0	Community	Well	Hospital/Health care
Florida	Nov	2013	*L. pneumophila* serogroup 1	ARI	4	4	0	Community	Other	Other^§§^
Florida	Apr	2014	*L. pneumophila* serogroup 1	ARI	2	2	0	Community	Well	Hotel/Motel/Lodge/Inn
Florida	Jun	2014	*L. pneumophila* serogroup 1	ARI	3	2	0	Community	Unknown	Long-term care facility
Florida	Aug	2014	*L. pneumophila* serogroup 1	ARI	6	4	0	Community	Unknown	Hotel/Motel/Lodge/Inn
Idaho	Sep	2014	*Giardia duodenalis*	AGI	2	0	0	Unknown	Unknown	Hotel/Motel/Lodge/Inn
Indiana	Jul	2013	*Cryptosporidium* sp.	AGI	7	0	0	Community	Unknown	Mobile home park
Indiana	Nov	2014	Unknown	AGI	3	0	0	Community	Unknown	Apartment/Condo
Kansas	Jun	2014	*L. pneumophila* serogroup 1	ARI	2	2	0	Community	Unknown	Hospital/Health care
Maryland	Nov	2012	*L. pneumophila* serogroup 1	ARI	2^¶¶^	2^¶¶^	0	Community	Well	Hotel/Motel/Lodge/Inn
Maryland	Feb	2013	Nitrite***	AGI, Neuro	14		0	Community	Lake/Reservoir/ Impoundment	Indoor workplace/Office
Maryland	Apr	2014	*L. pneumophila* serogroup 1	ARI	2	2	0	Community	Lake/Reservoir/ Impoundment	Apartment/Condo
Maryland	Jul	2014	*L. pneumophila* serogroup 1	ARI	2	1	0	Community	Well	Hotel/Motel/Lodge/Inn
Maryland	Aug	2014	*L. pneumophila* serogroup 1	ARI	2	2	0	Community	River/Stream	Prison/Jail (Juvenile/Adult)
Michigan	Jun	2014	*L. pneumophila* serogroup 1	ARI	45	45	7	Community	River/Stream	Hospital/Health care, Community/Municipality^†††^
Montana	Jul	2014	Norovirus GII.Pe-GII.4 Sydney	AGI	62	0	0	Transient, noncommunity	Well	Hotel/Motel/Lodge/Inn
New York	Jul	2013	*L. pneumophila* serogroup 1	ARI	2	2	0	Community	Lake/Reservoir/ Impoundment	Hospital/Health care
New York	Jun	2014	*L. pneumophila* serogroup 1	ARI	2	2	0	Community	Well	Hospital/Health care
North Carolina	Dec	2013	*L. pneumophila* serogroup 1	ARI	3	2	0	Community	Unknown	Long-term care facility
North Carolina	Dec	2013	*L. pneumophila* serogroup 1	ARI	7	3	0	Community	Unknown	Long-term care facility
North Carolina	May	2014	*L. pneumophila* serogroup 1	ARI	7	6	1	Community	Other	Long-term care facility
North Carolina	Jun	2014	*L. pneumophila* serogroup 1	ARI	3	3	0	Community	Unknown	Long-term care facility
North Carolina	Jul	2014	*L. pneumophila* serogroup 1	ARI	3	2	1	Community	Unreported	Long-term care facility
Ohio	Apr	2013	*L. pneumophila*	ARI	2	2	1	Unknown	Unknown	Long-term care facility
Ohio^§§§^	Sep	2013	Cyanobacterial toxin^¶¶¶^	AGI	6	0	0	Community	Lake/Reservoir/ Impoundment	Community/Municipality
Ohio	Jul	2014	*L. pneumophila* serogroup 1	ARI	14	4	0	Community	River/Stream	Long-term care facility
Ohio	Aug	2014	Cyanobacterial toxin^¶¶¶^	AGI	110			Community	Lake/Reservoir/ Impoundment	Community/Municipality
Ohio	Oct	2014	*Cryptosporidium* sp. (S)****	AGI	100	0	0	Individual	River/Stream	Farm/Agricultural setting
Ohio	Dec	2014	Viral, unknown (S)	AGI	2	0	0	Commercially bottled	Unknown	Private residence
Oregon	Jun	2013	*Cryptosporidium parvum* IIaA15G2R1	AGI	119	2	0	Community	Lake/Reservoir/ Impoundment	Community/Municipality
Oregon	Sep	2014	*L. pneumophila* serogroup 1	ARI	4	4	1	Community	Well	Apartment/Condo
Pennsylvania	Dec	2013	*L. pneumophila* serogroup 1	ARI	2	2	0	Unknown	Unknown	Hospital/Health care
Pennsylvania	Feb	2014	*L. pneumophila* serogroup 1	ARI	5	5	0	Community	River/Stream	Long-term care facility
Pennsylvania	Oct	2014	*L. pneumophila*	ARI	2	2	1	Community	Unknown	Long-term care facility
Rhode Island	Apr	2013	*L. pneumophila* serogroup 1	ARI	2	2	1	Community	Lake/Reservoir/ Impoundment	Hospital/Health care
Tennessee	Jul	2013	*Cryptosporidium parvum*	AGI	34	0	0	Transient, noncommunity^††††^	Spring	Camp/Cabin setting
Tennessee	Jun	2014	*Clostridium difficile* (S); *Escherichia coli*, Enteropathogenic (S)	AGI	12	0	0	Nontransient, noncommunity	Well	Camp/Cabin setting; Community/Municipality
Virginia	Jun	2013	*Cryptosporidium* sp.	AGI	19	0	0	Individual	Well	Farm/Agricultural setting
West Virginia	Jan	2014	4-Methylcyclo hexanemethanol (MCHM)^§§§§^	AGI	369	13	0	Community	River/Stream	Community/Municipality
Wisconsin	Aug	2014	*Giardia duodenalis*	AGI	3	0	0	Nontransient, noncommunity	Other	National forest
Wisconsin	Sep	2014	*Campylobacter jejuni*	AGI	5	0	0	Individual	Well	Private residence

**Abbreviations:** AGI = acute gastrointestinal illness; ARI = acute respiratory illness; *L. pneumophila* = *Legionella pneumophila*; Neuro = neurologic illnesses, conditions, or symptoms (e.g., meningitis); S = suspected.

* Etiologies listed are confirmed, unless indicated as suspected. For multiple-etiology outbreaks, etiologies are listed in alphabetical order.

^†^ The category of illness reported by ≥50% of ill respondents. All legionellosis outbreaks were categorized as ARI.

^§^ Value was set to “missing” in reports where zero hospitalizations were reported and the number of persons for whom information was available was also zero or for instances where reports are missing hospitalization data.

^¶^ Value was set to “missing” in reports where zero deaths were reported and the number of persons for whom information was available was also zero or for instances where reports are missing data on associated deaths.

** Community and noncommunity water systems are public water systems that have ≥15 service connections or serve an average of ≥25 residents for ≥60 days per year. A community water system serves year-round residents of a community, subdivision, or mobile home park. A noncommunity water system serves an institution, industry, camp, park, hotel, or business and can be nontransient or transient. Nontransient systems serve ≥25 of the same persons for ≥6 months of the year but not year-round (e.g., factories and schools) whereas transient systems provide water to places in which persons do not remain for long periods of time (e.g., restaurants, highway rest stations, and parks). Individual water systems are small systems not owned or operated by a water utility that have <15 connections or serve <25 persons.

^††^ Classification of all reported *Giardia* cases has changed from *Giardia intestinalis* to *Giardia duodenalis* to align with laboratory standards.

^§§^ Setting is listed as “other” because implicated facility houses both independent living and assisted living facilities.

^¶¶^ This count was not included in the analysis of the current report. This outbreak occurred in 2012 and was not reported in the previous drinking water outbreak report.

*** Patients’ methemoglobin levels ranged from 1.6% to 32.3%. Water was determined to be the source rather than food because all cases had direct exposure to water. Of the 14 cases, five used the water to make oatmeal or cream of wheat.

^†††^ This report includes both community and hospital-associated cases (27 of 45 patients reported health care/hospital exposure).

^§§§^ This is the first drinking water–associated outbreak of this etiology reported to the National Outbreak Reporting System.

^¶¶¶^ Microcystin was detected in finished water sampled from a community water system; levels exceeded state thresholds and resulted in a “Do not drink” advisory.

**** *Cryptosporidium* was detected in water samples but not in any clinical specimens.

^††††^ This system was registered as a community system as a result of the outbreak investigation.

^§§§§^ Illnesses were associated with exposure to 4-methylcyclohexanemethanol following a documented industrial spill into water supplying a public water system. However, individual levels of exposure could not be quantified in clinical specimens. Propylene glycol phenyl ether was also present in the spill at low concentrations.

The most commonly reported outbreak etiology was *Legionella* (57%), making acute respiratory illness the most common predominant illness type reported in outbreaks (Table 2). Thirty-five (83%) outbreaks were associated with public (i.e., regulated), community or noncommunity water systems,^¶^ and three (7%) were associated with unregulated, individual systems. Fourteen outbreaks occurred in drinking water systems with groundwater sources and an additional 14 occurred in drinking water systems with surface water sources. The most commonly cited deficiency, which led to 24** (57%) of the 42 drinking water–associated outbreaks, was the presence of *Legionella* in drinking water systems. In addition, 143 (14%) cases were associated with seven (17%) outbreak reports that had a deficiency classification indicating “unknown or insufficient information.”

**TABLE 2:** Rank order (most common to least common) of etiology, water system, water source, predominant illness, and deficiencies associated with 42 drinking water outbreaks and 1,006 outbreak-related cases of illness — United States, 2013–2014

Characteristic/Rank	Outbreaks (N = 42)		Cases (N = 1,006)	
	Category	No. (%)	Category	No. (%)
**Etiology**				
1	Bacteria,* Legionella*	24 (57.1)	Chemical/Toxin	499 (49.6)
2	Parasites	8 (19.1)	Parasites	289 (28.7)
3	Chemical/Toxin	4 (9.5)	Bacteria,* Legionella*	130 (12.9)
4	Viruses	3 (7.1)	Viruses	68 (6.8)
5	Bacteria, non-*Legionella*	1 (2.4)	Multiple bacteria	12 (1.2)
6	Multiple bacteria	1 (2.4)	Bacteria, non-*Legionella*	5 (0.5)
7	Unknown	1 (2.4)	Unknown	3 (0.3)
Water system*				
1	Community	30 (71.4)	Community	759 (75.4)
2	Noncommunity	5 (11.9)	Individual	124 (12.3)
3	Individual	3 (7.1)	Noncommunity	115 (11.4)
4	Unknown	3 (7.1)	Unknown	6 (0.6)
5	Bottled	1 (2.4)	Bottled	2 (0.2)
**Water source**				
1	Ground water	14 (33.3)	Surface water	795 (79.0)
2	Surface water	14 (33.3)	Ground water	157 (15.6)
3	Unknown	12 (28.6)	Unknown	39 (3.9)
4	Mixed^†^	1 (2.4)	Mixed	12 (1.2)
5	Unreported	1 (2.4)	Unreported	3 (0.3)
**Predominant illness** ^§^				
1	ARI	24 (57.1)	AGI	862 (85.7)
2	AGI	17 (40.5)	ARI	130 (12.9)
3	AGI; Neuro	1 (2.4)	AGI; Neuro	14 (1.4)
**Deficiency** ^¶^				
1	*Legionella* spp. in drinking water system**	23 (54.8)	Treatment not expected to remove contaminant	485 (48.2)
2	Unknown/Insufficient information^††^	7 (16.7)	Unknown/Insufficient information	143 (14.2)
3	Multiple^§§^	3 (7.1)	*Legionella* spp. in drinking water system	126 (12.5)
4	Treatment not expected to remove contaminant^¶¶^	3 (7.1)	Treatment deficiency	119 (11.8)
5	Untreated ground water***	3 (7.1)	Untreated ground water	70 (7.0)
6	Distribution system^†††^	1 (2.4)	Multiple	42 (4.2)
7	Premises plumbing system^§§§^	1 (2.4)	Premise plumbing system	14 (1.4)
8	Treatment deficiency^¶¶¶^	1 (2.4)	Distribution system	7 (0.7)

**Abbreviations:** AGI = acute gastrointestinal illness; ARI = acute respiratory illness; Neuro = neurologic illnesses, conditions, or symptoms (e.g., meningitis).

* Community and noncommunity water systems are public water systems that have ≥15 service connections or serve an average of ≥25 residents for ≥60 days per year. A community water system serves year-round residents of a community, subdivision, or mobile home park. A noncommunity water system serves an institution, industry, camp, park, hotel, or business and can be nontransient or transient. Nontransient systems serve ≥25 of the same persons for ≥6 months of the year but not year-round (e.g., factories and schools) whereas transient systems provide water to places in which persons do not remain for long periods of time (e.g., restaurants, highway rest stations, and parks). Individual water systems are small systems not owned or operated by a water utility that have <15 connections or serve <25 persons.

^†^ Includes outbreaks with mixed water sources (i.e., ground water and surface water).

^§^ The category of illness reported by ≥50% of ill respondents; all legionellosis outbreaks were categorized as ARI.

^¶^ Outbreaks are assigned one or more deficiency classifications. https://www.cdc.gov/healthywater/surveillance/deficiency-classification.html.

** Deficiency 5A. Drinking water, contamination of water at points not under the jurisdiction of a water utility or at the point of use: *Legionella* spp. in water system, drinking water.

^††^ Deficiency 99. Unknown/Insufficient information.

^§§^ Multiple deficiency classifications were assigned to three outbreaks. One outbreak had deficiency 2, 3 one had 3, 4, and one had 5a, 7 (deficiency in building/home-specific water treatment after the water meter or property line).

^¶¶^ Deficiency 13a. Current treatment processes not expected to remove a chemical contaminant: ground water.

*** Deficiency 2. Drinking water, contamination of water at/in the water source, treatment facility, or distribution system: untreated ground water.

^†††^ Deficiency 4. Drinking water, contamination of water at/in the water source, treatment facility, or distribution system: Distribution system deficiency, including storage (e.g., cross-connection, backflow, and contamination of water mains during construction or repair).

^§§§^ Deficiency 6. Drinking water, contamination of water at points not under the jurisdiction of a water utility or at the point of use; plumbing system deficiency after the water meter or property line (e.g., cross-connection, backflow, or corrosion products).

^¶¶¶^ Deficiency 3. Treatment deficiency (e.g., temporary interruption of disinfection, chronically inadequate disinfection, or inadequate or no filtration).

Among 1,006 cases attributed to drinking water–associated outbreaks, 50% of the reported cases were associated with chemical or toxin exposure, 29% were caused by parasitic infection (either *Cryptosporidium* or *Giardia*), and 13% were caused by *Legionella* infection (Table 2). Seventy-five percent of cases were linked to community water systems. Outbreaks in water systems supplied solely by surface water accounted for most cases (79%). Of the 1,006 cases, 86% originated from outbreaks in which the predominant illness was acute gastrointestinal illness. Three (7%) outbreaks in which treatment was not expected to remove the contaminant were associated with a chemical or toxin and resulted in 48% of all outbreak-associated cases.

## Discussion

Water treatment processes, regulations, and rapid response to illness outbreaks continue to reduce the transmission of pathogens, reduce exposure to chemicals and toxins, and protect the public drinking water supplies in the United States. Outbreaks reported during this surveillance period include the first reports of drinking water–associated outbreaks caused by harmful algal blooms as well as the continued challenges of preventing and controlling illnesses and outbreaks caused by *Legionella* and *Cryptosporidium*. Outbreaks in community water systems caused by chemical spills (West Virginia) (*2*), harmful algal blooms (Ohio), *Cryptosporidium* (Oregon) (*3*), and *Legionella* (Michigan) demonstrated that diverse contaminants can cause interruptions in water service, illnesses, and persistent community concern about drinking water quality. Outbreaks in community water systems can trigger large and complex public health responses because of their potential for causing communitywide illness and decreasing the availability of safe water for community members, businesses, and critical services (e.g., hospitals). These outbreaks highlight the importance of public health and water utility preparedness for emergencies related to contamination from pathogens, chemicals, and toxins.

*Legionella* continues to be the most frequently reported etiology among drinking water–associated outbreaks (*4*). All of the outbreak-associated deaths reported during this surveillance period as well as all of the outbreaks reported in hospital/health care settings or long-term care facilities, were caused by *Legionella*. A review of 27 Legionnaires’ disease outbreak investigations in which CDC participated during 2000–2014 identified at least one water system maintenance deficiency in all 23 investigations for which this information was available, indicating that effective water management programs in buildings at increased risk for *Legionella* growth and transmission (e.g., those with more than 10 stories or that house susceptible populations) can reduce the risk for Legionnaires’ disease (*5*,*6*). Although *Legionella* was detected in drinking water, multiple routes of transmission beyond ingestion of contaminated water more likely contributed to these outbreaks, such as aerosolization from domestic or environmental sources. *Cryptosporidium* was the second most common cause of both outbreaks and illnesses, demonstrating the continued threat from this chlorine-tolerant pathogen when drinking water supplies are contaminated. Existing drinking water regulations and filtration systems targeted to control *Cryptosporidium* help protect public health in community water systems that are primarily served by surface water sources or groundwater sources under the influence of surface water (*7*). Through the Epidemiology and Laboratory Capacity for Infectious Diseases (ELC) Cooperative Agreement, CDC has recently begun a laboratory-based cryptosporidiosis surveillance system in the United States, CryptoNet, to better track *Cryptosporidium* transmission and rapidly identify outbreak sources through molecular typing (*8*). The cyanobacterial toxin microcystin caused the largest reported toxin contamination of community drinking water in August 2013 and September 2014 and was responsible for extensive community and water disruptions. In June 2015, the Environmental Protection Agency released specific health advisory guidance for microcystin concentrations in drinking water (*9*). The contamination of a community drinking water supply with 4-metholcyclohexanementanol (MCHM) also illustrates the importance of source water protection from chemicals and toxins (*2*).

The findings in this report are subject to at least three limitations. First, 17% of drinking water–associated outbreak reports could not be assigned a specific deficiency classification other than “unknown or insufficient information,” because of a lack of information. Furthermore, the deficiency classification most frequently reported (“presence of *Legionella* in drinking water systems”) does not provide insight into the specific factors contributing to *Legionella* amplification and transmission. Second, the detection and investigation of outbreaks might be incomplete. Because of universal exposure to water, linking illness to drinking water is inherently difficult through traditional outbreak investigation methods (e.g., case-control and cohort studies) (*10*). Finally, reporting capabilities and requirements vary among states and localities. Therefore, outbreak surveillance data likely underestimate actual occurrence of outbreaks and should not be used to estimate the actual number of outbreaks or cases of waterborne disease.

Public health surveillance is necessary to detect waterborne disease and outbreaks, and to continue to monitor health trends associated with drinking water exposure. Despite resource constraints, 19 states reported drinking water–associated outbreaks for 2013–2014 compared with 14 for the previous reporting period (*4*). In this reporting cycle, more reported outbreaks and cases were caused by parasites and chemicals than by non-*Legionella* bacteria, and more cases were reported from community systems than from individual systems. Most of the outbreaks and illnesses reported in this period were in community systems, which serve larger numbers of persons; outbreaks in these systems can sicken entire communities. Although individual, private water systems likely serve fewer persons than community systems, they can still result in relatively large numbers of illnesses. One outbreak reported during 2013–2014 in an individual system led to 100 estimated illnesses associated with a wedding. The public health challenges highlighted here underscore the need for rapid detection, identification of the cause, and response when drinking water is contaminated by infectious pathogens, chemicals, or toxins to prevent and control waterborne illness and outbreaks.

**Summary**

**What is already known about this topic?**

Waterborne disease and outbreaks associated with drinking water continue to occur in the United States. CDC collects data on waterborne disease outbreaks submitted from all states and territories through the National Outbreak Reporting System.

**What is added by this report?**

During 2013–2014, a total of 42 drinking water–associated outbreaks were reported to CDC, resulting in at least 1,006 cases of illness, 124 hospitalizations, and 13 deaths. *Legionella* was responsible for 57% of outbreaks and 13% of illnesses, and chemicals/toxins and parasites together accounted for 29% of outbreaks and 79% of illnesses. Eight outbreaks caused by parasites resulted in 289 (29%) cases, among which 279 (97%) were caused by *Cryptosporidium* and 10 (3%) were caused by *Giardia duodenalis*. Chemicals or toxins were implicated in four outbreaks involving 499 cases, with 13 hospitalizations, including the first outbreaks associated with algal toxins.

**What are the implications for public health practice?**

Continued public health surveillance is necessary to detect waterborne disease and monitor health trends associated with drinking water exposure. When drinking water is contaminated by infectious pathogens, chemicals, or toxins, public health agencies need to provide rapid detection, identification of the cause, and response to prevent and control waterborne illness and outbreaks. Effective water management programs in buildings at increased risk for *Legionella* growth and transmission can reduce the risk for disease from drinking water pathogens.

## Footnotes

* Outbreak reports can be submitted by public health agencies in the U.S states, District of Columbia, Guam, Puerto Rico, Marshall Islands, Federated States of Micronesia, Northern Mariana Islands, Palau, and U.S. Virgin Islands.

† Drinking water, also called potable water, is water for human consumption (e.g., drinking, bathing, showering, hand-washing, teeth brushing, food preparation, dishwashing, and maintaining oral hygiene) and includes water collected, treated, stored, or distributed in public and individual water systems, as well as bottled water.

§ Waterborne disease outbreaks are assigned one or more deficiency classifications based on available data. The deficiencies provide information regarding how the water became contaminated, characteristics of the water system, and factors leading to waterborne disease outbreaks. Outbreaks are assigned one or more deficiency classifications based on available data. https://www.cdc.gov/healthywater/surveillance/deficiency-classification.html.

¶ Community and noncommunity water systems are public water systems that have ≥15 service connections or serve an average of ≥25 residents for ≥60 days per year. A community water system serves year-round residents of a community, subdivision, or mobile home park. A noncommunity water system serves an institution, industry, camp, park, hotel, or business and can be nontransient or transient. Nontransient systems serve ≥25 of the same persons for ≥6 months of the year but not year-round (e.g., factories and schools) whereas transient systems provide water to places in which persons do not remain for long periods of time (e.g., restaurants, highway rest stations, and parks). Individual water systems are small systems not owned or operated by a water utility that have <15 connections or serve <25 persons.

** One of the 24 outbreaks included both deficiencies 5a and 7 under the “multiple” classification.
